# Immune-Ageing Evaluation of Peripheral T and NK Lymphocyte Subsets in Chinese Healthy Adults

**DOI:** 10.1007/s43657-023-00106-0

**Published:** 2023-05-23

**Authors:** Zhenghu Jia, Zhiyao Ren, Dongmei Ye, Jiawei Li, Yan Xu, Hui Liu, Ziyu Meng, Chengmao Yang, Xiaqi Chen, Xinru Mao, Xueli Luo, Zhe Yang, Lina Ma, Anyi Deng, Yafang Li, Bingyu Han, Junping Wei, Chongcheng Huang, Zheng Xiang, Guobing Chen, Peiling Li, Juan Ouyang, Peisong Chen, Oscar Junhong Luo, Yifang Gao, Zhinan Yin

**Affiliations:** 1 Guangdong Provincial Key Laboratory of Tumor Interventional Diagnosis and Treatment, Zhuhai Institute of Translational Medicine Zhuhai People’s Hospital Affiliated With Jinan University, Jinan University, Zhuhai, 519000 Guangdong China; 2 The Biomedical Translational Research Institute, Health Science Center (School of Medicine), Jinan University, Guangzhou, 510632 Guangdong China; 3 Guangzhou Purui Biotechnology Co., Ltd., Guangzhou, 510660 Guangdong China; 4 Department of Systems Biomedical Sciences, School of Medicine, Jinan University, Guangzhou, 510632 Guangdong China; 5 Guangzhou Geriatric Hospital, Guangzhou, 510550 Guangdong China; 6 Organ Transplantation Center, The First Affiliated Hospital, Sun Yat-sen University, Guangzhou, 510080 Guangdong China; 7 Emergency Department, The First Affiliated Hospital of Jinan University, Jinan University, Guangzhou, 510632 Guangdong China; 8 NHC Key Laboratory of Hormones and Development, Tianjin Key Laboratory of Metabolic Diseases, Chu Hsien-I Memorial Hospital, Tianjin Medical University, Tianjin, 300134 China; 9 Tianjin Institute of Endocrinology, Tianjin Medical University, Tianjin, 300134 China; 10 Zhongke Regenerative Medicine Technology Co., Ltd, Dongguan, 523808 Guangdong China; 11 Wuhan Purui Medical Laboratory Co., Ltd, Wuhan, 430223 Hubei China; 12 Department of Clinical Laboratory, Department of Laboratory Medicine, The First Affiliated Hospital of Sun Yat-sen University, Guangzhou, 510080 Guangdong China

**Keywords:** Immunosenescence, Phenotype, T cell subset, Natural killer cell subsets, Immune-ageing modelling

## Abstract

Ageing is often accompanied with a decline in immune system function, resulting in immune ageing. Numerous studies have focussed on the changes in different lymphocyte subsets in diseases and immunosenescence. The change in immune phenotype is a key indication of the diseased or healthy status. However, the changes in lymphocyte number and phenotype brought about by ageing have not been comprehensively analysed. Here, we analysed T and natural killer (NK) cell subsets, the phenotype and cell differentiation states in 43,096 healthy individuals, aged 20–88 years, without known diseases. Thirty-six immune parameters were analysed and the reference ranges of these subsets were established in different age groups divided into 5-year intervals. The data were subjected to random forest machine learning for immune-ageing modelling and confirmed using the neural network analysis. Our initial analysis and machine modelling prediction showed that naïve T cells decreased with ageing, whereas central memory T cells (Tcm) and effector memory T cells (Tem) increased cluster of differentiation (CD) 28-associated T cells. This is the largest study to investigate the correlation between age and immune cell function in a Chinese population, and provides insightful differences, suggesting that healthy adults might be considerably influenced by age and sex. The age of a person's immune system might be different from their chronological age. Our immune-ageing modelling study is one of the largest studies to provide insights into ‘immune-age’ rather than ‘biological-age’. Through machine learning, we identified immune factors influencing the most through ageing and built a model for immune-ageing prediction. Our research not only reveals the impact of age on immune parameter differences within the Chinese population, but also provides new insights for monitoring and preventing some diseases in clinical practice.

**Supplementary Information:**

The online version contains supplementary material available at 10.1007/s43657-023-00106-0.

## Introduction

Ageing is a complex process that involves numerous physiological and biological changes. It greatly impacts the immune system, leading to alterations in immune function and subtypes. Over time, the ability of the immune system to respond to foreign pathogens and infections, and their capability to recall and refine memory responses slowly diminishes.

T cells are one of the most important subsets in the immune system and they play a critical role in the development of age-related immune impairment. Mittelbrunn and Kroemer recently proposed 10 hallmarks of T cell ageing (Mittelbrunn and Kroemer 2021). Amongst them are the phenotypic changes, which include naïve-memory T cell imbalance, T cell receptor (TCR) repertoire reduction, T cell senescence, and the lack of effector plasticity. Another process often observed during ageing is T cell exhaustion. It is the accumulation of dysfunctional, terminally differentiated T cells and is often observed in the ageing immune system due to long-term exposure to foreign/viral antigen stimulation (Gustafson 2021; Huang et al. 2021). It is typically caused by chronic infection or the development of cancer. It differs from T cell senescence in terms of phenotypic and functional features, such as surface molecules and cytokines (Nie et al. 2022). Notably, T cell exhaustion is often caused by disease-associated immune dysfunction, and T cell senescence is usually caused by biological ageing (Mogilenko et al. 2021a).

Natural killer (NK) cells are another subset of immune cells that are greatly affected during the ageing process (Solana et al. 2014). Several researchers have investigated the changes in NK cells during the ageing process; however, the results are ambiguous (Choi et al. 2014; Qin et al. 2016; Valdiglesias et al. 2015). This might be because the study population and control groups selected in these studies were distinctive. In the Caucasian cohort, the percentage of NK cells remained constant in ageing individuals, whereas in two Asians studies, the number of NK cells was found to increase with ageing. Another study performed by a UK group suggests that the absolute number of NK cells remained unchanged during the ageing process whereas that of CD56^bright^ NK cells declined with increasing age (Chidrawar et al. 2006).

Studies have focussed primarily on the phenotypic changes in immune subsets in disease-related settings (Carrasco et al. 2022; Dizaji Asl et al. 2021). However, the comparison is usually made when using a small control cohort or when there is a lack of appropriate age-related controls. The reference range for T and NK subsets in different age groups, in both clinical research and clinical practice monitoring, varied greatly in different studies. This is extremely important in the clinical setting as different diseases are more likely to occur in different age groups.

Thus, we investigated the phenotype of T and NK subsets, including differentiation states and exhaustion markers in more than 70,000 different individuals, aged between 20 and 90 years, with an aim to establish the reference value for these subsets in different age groups. We also aimed to build an immune age prediction model.

## Materials and Methods

### Study Design and Participants

The study population included 76,477 individuals who underwent blood tests conducted by Purui Laboratory testing (Wuhan, China) through 07/2020-05/2021. It was made accessible by the Department of Translation Studies, Jinan University and annotated with 36 peripheral T and NK blood subtypes. Amongst the 76,477 cases, 65,532 individuals were declared to be healthy during the physical check (The exclusion criteria included: family history of genetic disorder; cardiovascular and cerebrovascular disease; respiratory system disease; digestive system disease; immune system disorder). Outliers were removed from the mean plus or minus three standard deviations (± 3 × SD) in each age group, and 43,096 healthy individuals remained for further analysis. These samples were further divided into the following 11 groups divided by 5-year intervals. Samples from both male and female individuals were included in each age group. Any test subject with percentage values of any immune cell subtype greater than the mean ± 3 × SD was considered an outlier and the corresponding donor was removed.

### Sample Preparation

Five millilitre of venous blood was collected in a heparin sodium anticoagulant tube. The blood samples were mixed gently and immediately after collection to prevent clotting. Violent mixing of the samples was avoided to prevent haemolysis. The samples were checked to ensure that they meet the requirements, including the absence of clots and haemolysis, blood sample volume, sample barcode, and basic information. Qualified samples were selected for subsequent experiments. Samples were tested in a timely manner after collection and were stored at room temperature for no more than 48 h.

### Flow Cytometry Analysis

The antibody MIX was first configured for the experiment; it included phycoerythrin (PE) anti-human Vδ2 (clone: B6, cat No: 555739, BD Biosciences), APC-Cy7 anti-human CD4 (clone: RPA-T4, cat No: 557871, BD Biosciences), PE-Cy7 anti-human CD45RA (clone: HI100, cat No: 560675, BD Biosciences), allophycocyanin (APC) anti-human CD57 (clone: NK-1, cat No: 560845, BD Biosciences), BB515 anti-human CC-chemokine receptor 7 (CCR7) (clone: 3D12, cat No: 565869, BD Biosciences), BB700 anti-human CD56 (clone: B159, cat No: 566400, BD Biosciences), R718 anti-human CD3 (clone: UCHT1, cat No: 566953, BD Biosciences), BV510 anti-human CD28 (clone: L293, cat No: 742526, BD Biosciences), BV605 anti-human programmed cell death protein 1 (PD-1) (clone: EH12.2H7, cat No: 329924, BD Biosciences), BV421 anti-human TCR γδ (clone: B1, cat No: 331218, BD Biosciences), batch-to-batch variation was performed when changes. Then 100 μL venous blood and 10 μL antibody MIX were added to the flow tubes, which were shaken gently and incubated in the dark for 15 min. After incubation, 800 μL red blood cell lysis buffer (Solarbio, Beijing, China) was added to the tubes for lysis at room temperature for 15 min. After lysis, 2 mL of phosphate-buffered saline (PBS, Solarbio, Beijing, China) was added to stop the lysis, and the tubes were shaken gently and centrifuged at 1000 rpm for 5 min. Finally, the resulting cell pellets were resuspended in 350 μL of PBS. The fluorescence of different cell subsets was detected using BD LSRFortessa flow cytometry. The gating strategy is shown in Supplementary Fig. 1.

### Machine Learning

Random forest (RF) machine-learning method was used to build an immune age prediction model. Healthy donors were classified as young or old according to their chronological age (young: 20–29 year-old, old: ≥ 65 year-old) for model training. Random sampling for training and cross-validation was repeated 10 times, and the averaged results were used to reduce potential variability. The predictive ability was recorded based on the area under curve (AUC) value using the receiver operating characteristic (ROC) analysis. The mid-age healthy (30–64 year-old) and unhealthy cohorts (20–88 year-old, with recorded chronic diseases) were introduced into the model for immune age group prediction (i.e. young or old). The neural network deep-learning approach was used similarly to validate the results from the RF machine-learning model. The importance of each feature (immune cell subtype percentage) was inferred from the machine-learning prediction model and linear regression.

### Statistical Analysis

All statistics were performed with R (v4.1.0) and Python (v3.8.0). *p* values less than 0.05 (Student’s *t* test, two tailed) were considered statistically significant.

## Results

### Reference Values for Peripheral T and NK Lymphocyte Subsets were Established

**Fig. 1:**
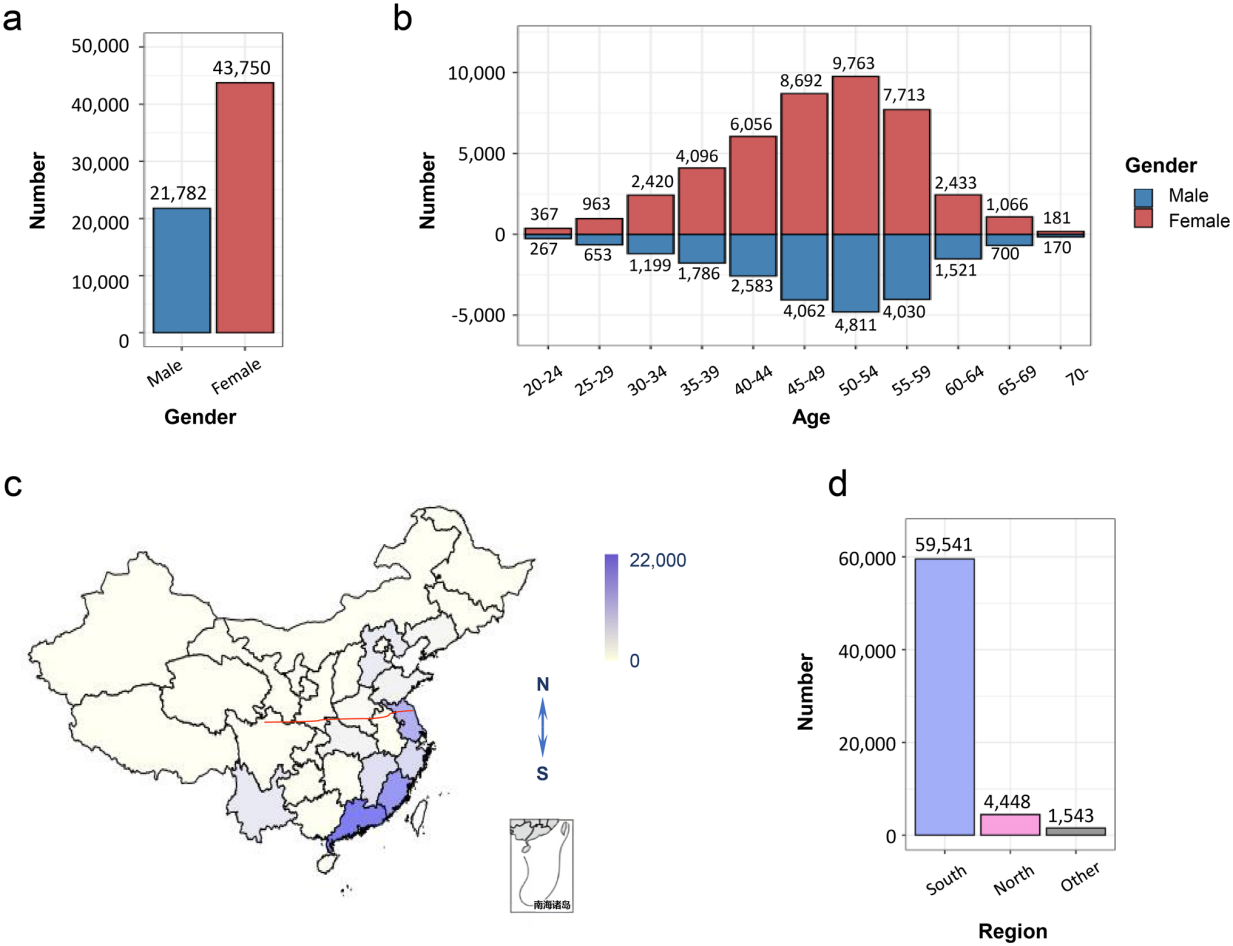
Delineation of the gender, age stages, and geographical distribution of 65,532 healthy Chinese donors. **a** Histogram of donor gender. **b** Histogram of donor age by 5-years interval. **c** Geological distribution of donors at the time of sample collection. The red line represents the north–south climate demarcation line of China. **d** Histogram of donors by sample collection location. The provinces located above and below the north–south climate demarcation line were considered as North and South, respectively. The donors without province information were defined as ‘Other’

We analysed 65,532 healthy individuals and established reference values for peripheral T and NK lymphocyte subsets, including T cells, CD4^+^ T cells, CD8^+^ T cells, CD56^+^ NK cells, CD3^+^CD56^+^ T natural killer (TNK) cells, γδT, Vδ2, naïve CD4 T cells, terminal differentiated CD4 T cells, central memory CD4 T cells, effector T cells, naïve CD8 T cells, terminal differentiated CD8 T cells, central memory CD8 T cells, CD57-associated subsets, CD28-associated subsets, and PD-1-associated subsets in each age group (Table 1). Amongst the 65,532 healthy individuals, the number of male and female individuals were 21,782 and 43,750 accordingly; the ratio of male to female was roughly 1:2 (Fig. 1a). The detailed analysis of our cohort is shown in Fig. 1b,d.

**Table 1:** Reference values for different immune subsets across age groups

	Age groups (year-old)										
	20–24	25–29	30–34	35–39	40–44	45–49	50–54	55–59	60–64	65–69	70
CD3+ (%lymphocyte)	68.72 ± 9.63	69.62 ± 9.62	70.28 ± 9.80	69.94 ± 9.88	69.82 ± 9.89	69.34 ± 10.03	69.06 ± 9.99	68.78 ± 9.98	68.14 ± 9.93	67.10 ± 9.93	67.13 ± 9.95
CD3+ CD4+ (% CD3+)	48.84 ± 12.87	49.43 ± 13.39	50.42 ± 13.54	51.74 ± 13.67	52.77 ± 14.11	53.46 ± 14.20	54.38 ± 14.39	54.06 ± 14.54	53.59 ± 14.94	53.45 ± 14.84	52.92 ± 15.86
CD3+ CD8+ (% CD3+)	50.74 ± 12.95	50.18 ± 13.41	49.10 ± 13.54	47.73 ± 13.70	46.71 ± 14.15	46.02 ± 14.22	45.12 ± 14.38	45.50 ± 14.53	46.06 ± 14.90	46.22 ± 14.86	46.71 ± 15.85
CD3-CD56+ (%lymphocyte)	8.45 ± 5.83	7.84 ± 5.47	7.39 ± 5.19	7.50 ± 5.35	7.74 ± 5.49	7.88 ± 5.76	8.01 ± 5.89	8.36 ± 6.16	8.98 ± 6.44	9.63 ± 6.80	10.45 ± 7.22
CD3+ CD56+ (% CD3+)	5.54 ± 4.14	5.66 ± 4.24	5.47 ± 4.12	5.16 ± 3.84	5.20 ± 3.89	5.07 ± 3.89	4.92 ± 3.89	4.86 ± 3.97	4.78 ± 4.05	4.65 ± 3.76	4.56 ± 3.80
CD3+ γδT+ (% CD3+)	7.67 ± 4.74	7.84 ± 5.29	7.01 ± 4.73	6.63 ± 4.65	6.29 ± 4.52	6.00 ± 4.39	5.73 ± 4.32	5.62 ± 4.31	42 ± 4.21	5.30 ± 4.05	4.88 ± 4.04
CD3+ γδT+ Vδ2+ (% γδT)	57.07 ± 23.51	54.98 ± 24.06	54.00 ± 24.33	51.76 ± 24.47	50.70 ± 24.65	49.61 ± 24.57	49.00 ± 25.13	49.73 ± 25.56	49.79 ± 25.75	47.29 ± 25.97	46.16 ± 25.63
CD3+ CD4+ CD45RA+ CCR7+ (% CD4+)	37.75 ± 13.85	36.16 ± 13.27	34.76 ± 13.77	33.52 ± 13.71	32.16 ± 13.56	31.23 ± 13.49	31.28 ± 13.80	31.41 ± 14.13	31.72 ± 14.64	31.2 ± 14.72	29.54 ± 13.83
CD3+ CD4+ CD45RA+ CCR7– (% CD4 +)	9.50 ± 5.96	9.19 ± 5.61	9.03 ± 6.09	8.86 ± 5.90	8.84 ± 5.87	8.76 ± 5.96	8.66 ± 6.05	8.48 ± 6.06	8.49 ± 6.26	8.69 ± 6.38	9.05 ± 7.21
CD3+ CD4+ CD45RA-CCR7+ (% CD4 +)	19.57 ± 6.34	20.99 ± 6.94	21.47 ± 7.38	22.21 ± 7.86	22.83 ± 8.02	23.12 ± 8.14	23.45 ± 8.59	23.70 ± 8.66	23.67 ± 8.57	23.77 ± 8.81	24.32 ± 8.79
CD3+ CD4+ CD45RA-CCR7– (% CD4+)	33.16 ± 11.42	33.66 ± 11.58	34.75 ± 12.14	35.40 ± 12.2	36.18 ± 12.58	36.88 ± 12.92	36.61 ± 13.12	36.41 ± 13.39	36.12 ± 13.78	36.33 ± 13.84	37.09 ± 13.60
CD3+ CD8+ CCR7+ CD45RA+ (% CD8 +)	29.76 ± 16.25	30.18 ± 16.13	28.00 ± 15.29	26.44 ± 14.72	23.84 ± 13.62	21.3 ± 12.97	19.57 ± 12.18	17.87 ± 11.59	15.92 ± 10.36	14.07 ± 9.39	11.96 ± 7.93
CD3+ CD8+ CCR7-CD45RA+ (% CD8+)	33.48 ± 16.92	34.07 ± 16.25	35.09 ± 16.34	35.84 ± 16.10	37.31 ± 16.51	38.84 ± 16.91	40.01 ± 17.14	41.54 ± 17.42	42.47 ± 17.65	44.18 ± 17.07	44.97 ± 17.30
CD3+ CD8+ CCR7+ CD45RA– (% CD8 +)	3.47 ± 2.46	3.57 ± 2.44	3.81 ± 2.55	4.08 ± 2.84	4.38 ± 3.00	4.59 ± 3.20	4.82 ± 3.44	4.90 ± 3.58	5.11 ± 3.69	5.15 ± 3.70	5.32 ± 3.86
CD3+ CD8+ CCR7-CD45RA– (% CD8+)	33.30 ± 16.69	32.19 ± 15.99	33.11 ± 15.94	33.64 ± 15.96	34.47 ± 16.13	35.28 ± 16.25	35.60 ± 16.54	35.69 ± 16.61	36.50 ± 17.12	36.61 ± 16.76	37.75 ± 16.71
CD3-CD56+ CD57+ (% CD3-CD56+)	37.80 ± 22.12	38.17 ± 22.63	37.11 ± 23.08	36.94 ± 23.24	36.71 ± 23.46	36.59 ± 23.61	35.41 ± 24.05	35.62 ± 23.65	35.97 ± 23.78	38.10 ± 23.90	38.12 ± 25.43
CD3+ CD56+ CD57+ (% CD3+ CD56+)	26.10 ± 17.91	28.09 ± 18.51	28.11 ± 19.08	28.93 ± 19.47	30.76 ± 20.43	31.78 ± 20.77	31.91 ± 21.22	32.53 ± 21.66	32.93 ± 21.68	35.60 ± 22.40	34.35 ± 22.84
CD3+ γδ+ Vδ2+ CD57+ (%Vδ2+)	16.65 ± 18.09	17.47 ± 18.67	16.51 ± 17.98	16.81 ± 18.87	16.89 ± 19.25	16.63 ± 19.00	16.20 ± 18.94	17.12 ± 20.25	17.88 ± 21.13	19.49 ± 22.59	18.06 ± 19.69
CD3+ CD4+ CD28+ (% CD4+)	92.08 ± 7.20	91.74 ± 6.88	90.53 ± 7.66	90.35 ± 7.88	89.29 ± 8.67	88.65 ± 8.99	88.46 ± 9.09	88.10 ± 9.39	87.26 ± 10.18	86.86 ± 10.39	85.26 ± 11.74
CD3+ CD4+ CD28- (% CD4+)	7.83 ± 7.11	8.23 ± 6.86	9.45 ± 7.63	9.62 ± 7.86	10.68 ± 8.65	11.32 ± 8.97	11.50 ± 9.08	11.87 ± 9.37	12.71 ± 10.17	13.13 ± 10.37	14.70 ± 11.71
CD3+ CD4+ CD28-CD57+ (% CD4+)	4.02 ± 4.54	4.06 ± 4.16	4.69 ± 4.91	4.58 ± 4.66	5.28 ± 5.38	5.67 ± 5.70	5.62 ± 5.70	5.75 ± 5.87	6.23 ± 6.29	6.79 ± 6.67	7.84 ± 7.95
CD3+ CD4+ CD28-PD-1+ (% CD4+)	0.16 ± 0.30	0.14 ± 0.26	0.13 ± 0.26	0.12 ± 0.25	0.13 ± 0.28	0.13 ± 0.28	0.12 ± 0.26	0.13 ± 0.28	0.17 ± 0.36	0.15 ± 0.29	0.18 ± 0.32
CD3+ CD8+ CD28+ (% CD8+)	62.01 ± 16.29	60.65 ± 15.32	59.9 ± 15.84	59.17 ± 15.87	57.50 ± 16.50	56.04 ± 16.89	54.22 ± 17.04	52.58 ± 17.16	50.21 ± 17.50	48.70 ± 17.01	48.59 ± 17.69
CD3+ CD8+ CD28- (% CD8+)	37.96 ± 16.30	39.32 ± 15.31	40.08 ± 15.84	40.81 ± 15.86	42.49 ± 16.50	43.94 ± 16.89	45.77 ± 17.04	47.41 ± 17.16	49.79 ± 17.51	51.28 ± 17.02	51.39 ± 17.69
CD3+ CD4-CD28-CD57+ (% CD4-)	16.42 ± 12.31	17.02 ± 12.14	16.84 ± 12.87	16.96 ± 12.65	18.25 ± 13.60	19.03 ± 14.05	19.06 ± 14.18	19.48 ± 14.55	20.48 ± 14.99	22.03 ± 15.41	20.41 ± 15.10
CD3+ CD4-CD28-PD-1+ (% CD4-)	0.65 ± 0.86	0.68 ± 0.90	0.67 ± 0.98	0.63 ± 0.92	0.65 ± 0.99	0.63 ± 0.98	0.60 ± 0.93	0.60 ± 0.94	0.63 ± 0.96	0.58 ± 0.93	0.60 ± 1.05
CD3+ CD4+ PD-1+ (% CD4+)	5.05 ± 5.56	5.70 ± 6.18	5.45 ± 6.07	5.76 ± 6.56	5.44 ± 6.26	5.31 ± 6.13	5.07 ± 5.98	4.77 ± 5.63	4.63 ± 5.43	4.36 ± 5.01	4.00 ± 4.73
CD3+ CD4-CD28+ PD-1+ (% CD4-)	3.90 ± 3.62	4.20 ± 3.72	4.02 ± 3.71	4.10 ± 3.90	3.97 ± 3.78	3.89 ± 3.79	3.70 ± 3.65	3.42 ± 3.40	3.41 ± 3.46	3.13 ± 3.32	2.96 ± 3.09
CD3+ γδ+ Vδ2+ PD-1+ (%Vδ2+)	5.10 ± 5.66	5.69 ± 6.65	5.29 ± 6.25	5.56 ± 6.40	5.30 ± 6.39	5.37 ± 6.71	5.05 ± 6.31	4.87 ± 6.29	4.83 ± 5.92	4.44 ± 5.81	4.51 ± 5.58
CD3-CD56+ PD-1+ (%CD3-CD56+)	1.11 ± 1.49	1.16 ± 1.60	1.13 ± 1.57	1.18 ± 1.69	1.28 ± 1.94	1.22 ± 1.79	1.10 ± 1.62	1.02 ± 1.45	1.07 ± 1.57	1.04 ± 1.47	1.00 ± 1.42
CD3 + CD56 + PD-1 + (%CD3 + CD56 +)	5.50 ± 6.20	5.04 ± 5.36	4.93 ± 5.65	4.84 ± 5.35	4.56 ± 5.15	4.3 ± 4.98	3.94 ± 4.59	3.78 ± 4.59	3.88 ± 4.6	3.44 ± 4.11	3.44 ± 3.99
CD3+ CD4+ CD28-CD57+ PD-1+ (% CD4 +)	0.35 ± 1.44	0.49 ± 1.62	0.47 ± 1.58	0.40 ± 1.32	0.49 ± 1.56	0.42 ± 1.30	0.40 ± 1.25	0.46 ± 1.44	0.60 ± 1.64	0.62 ± 1.75	0.74 ± 2.23
CD3+ CD4-CD28-CD57+ PD-1+ (% CD4-)	0.71 ± 1.27	0.87 ± 1.56	0.82 ± 1.55	0.78 ± 1.53	0.81 ± 1.61	0.79 ± 1.63	0.71 ± 1.47	0.74 ± 1.60	0.81 ± 1.61	0.66 ± 1.21	0.67 ± 1.19
CD3+ γδ+ Vδ2+ CD57+ PD-1+ (%Vδ2+)	0.87 ± 1.24	1.12 ± 1.72	1.16 ± 1.82	1.23 ± 2.00	1.28 ± 2.08	1.25 ± 2.11	1.25 ± 2.11	1.21 ± 2.03	1.21 ± 2.04	1.18 ± 2.23	0.94 ± 1.79
CD3-CD56+ CD57+ PD-1+ (%CD3-CD56+)	0.28 ± 0.43	0.36 ± 0.56	0.37 ± 0.62	0.36 ± 0.60	0.41 ± 0.68	0.43 ± 0.73	0.40 ± 0.67	0.41 ± 0.69	0.37 ± 0.63	0.37 ± 0.63	0.29 ± 0.52
CD3 + CD56 + CD57 + PD-1+ (%CD3+ CD56+)	0.96 ± 1.36	1.23 ± 1.78	1.48 ± 2.37	1.39 ± 2.10	1.50 ± 2.25	1.49 ± 2.30	1.54 ± 2.49	1.54 ± 2.54	1.41 ± 2.32	1.18 ± 1.93	1.38 ± 2.37

Notably, when we further divided the population according to climate demarcation line of China, 30 immune cell subsets showed significant differences between the North and South region. The detailed differences amongst these immune cells are shown in Supplementary Table 1.

### The Proportion of T Cells was Relatively Stable in Different Age Groups

T cells are derived from haematopoietic stem cells in the bone marrow, and their precursors are transported to the thymus for maturation. T cells play an important role in cellular immune responses.

The proportion of T cells was relatively stable in different age groups. The inflections between the lowest and highest was in the range of 67.1–70.3 (within 95% interval). The highest percentage of T cells appeared between the ages 30 and 34, and the lowest percentage of T cells occurred at ≥ 65 years of age (Table 1). Both males and females shared a similar trend in T cells through ageing, even though the percentage of T cells was higher in males than in females (Supplementary Fig. 2).

Based on T cell co-receptor expression, T cells can be further divided into CD4 and CD8 T cells. After receiving antigen stimulation, naïve CD4^+^ T cells proliferate and differentiate into functional CD4^+^ T cells, which can produce cytokines and transmit signals to the immune system to initiate immune responses. CD8^+^ T cells can specifically kill antigen-expressed target cells by producing IFN-γ, TNF-α, and granzymes, which participate in the varied response to foreign pathogens (Jansen et al. 2019). We found that, in our cohort, CD4 T cells tended to increase with age, whereas CD8 T cells showed a decreasing trend (Table 1). Notably, females tend to have a higher percentage of the CD8 population than males in all age groups (Supplementary Fig. 2). The opposite occurred in the CD4 population, where males had a higher percentage (Supplementary Fig. 2).

### Dynamic Changes in CD8 T Cells Subsets in Peripheral Blood Through Ageing

**Fig. 2:**
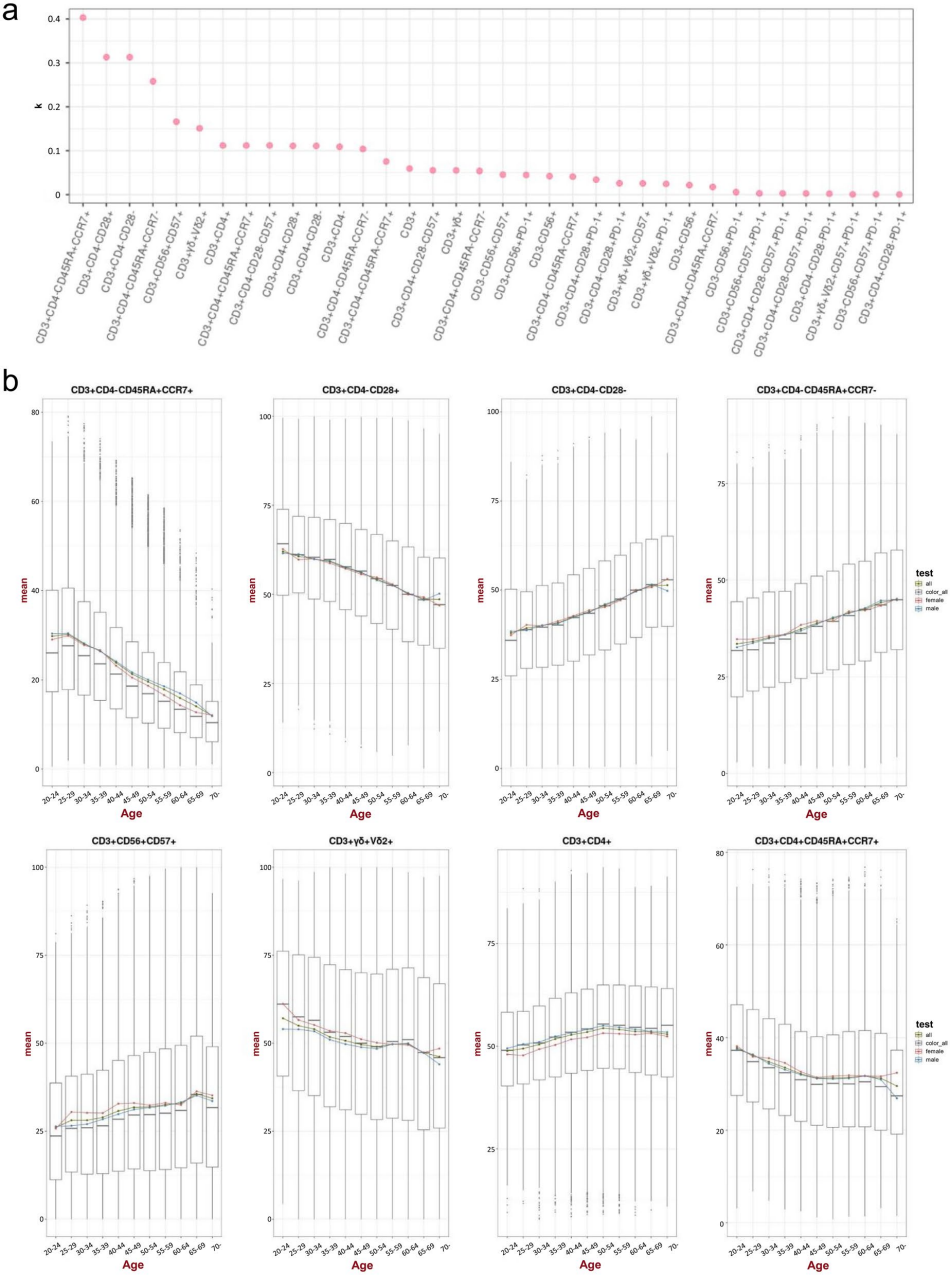
Dynamics of 36 immune cell subtype population through healthy ageing. **a** Absolute value of slope (*k*) for population size-age linear regression of each immune cell subtype. Higher value implies higher correlation (or anti-correlation) with age. **b** Boxplots of representative immune cell subtype population size across different age intervals. The red, blue, and green dots represent the mean values for female, male, and all donors, respectively. For the boxplots, the outlines of the boxes represent the first and third quartiles. The line inside each box represents the median, and boundaries of the whiskers are found within the 1.5 × IQR value, with dots representing outliers

We further analysed the changes in the 36 immune subsets in different age groups (Fig. 2a). As expected, naïve CD8 T cells showed the most significant changes in the ageing process with a K_*slope*_ = 0.4. Other than naïve CD8 T cells, functional CD8 T cells and terminally differentiated CD8 T cells were also found to have a strong association with ageing. CD8^+^ T cells are involved in the mediation of cell–cell interactions within the immune system, and participate in the processes of antitumor immunity, antiviral infection, and graft-versus-host reaction (Jansen et al. 2019). The impact of ageing on CD8 T cells might suggest the effect the immune system has against foreign pathogens, which lowered through ageing. Notably, similar effect was not found in CD4 subtypes. CD4 subtypes tend to have a rather stable trend in different age groups. Remarkably, no changes were noted for PD-1-associated subsets in the different age groups, which might suggest PD-1 is an immune check point marker that is not related to the ageing process. Detailed trend lines of the top eight ageing-associated immune subtypes in our analysis are shown in Fig. 2b.

### Ageing Greatly Impacts the Innate T cell Population

Innate T cells, as a bridge between innate and adaptive immune responses, show unique biological functions and clinical application value (Vantourout and Hayday 2013). Amongst the 36 immune subsets, γδ T and TNK cells belong to the innate T cell population. The age inflection point of the γδ T cell function was approximately 35 years. The proportion of γδ T cells decreased with age after 35 years (Supplementary Fig. 2). A similar pattern was observed for TNK cells, with a decline in cell percentage from 35 years onwards (Supplementary Fig. 2).

In humans, two major types of γδ T cells are characterised as either Vδ1 or Vδ2 T cells, depending on the δ chain expressed. The Vδ1 population mainly resides in the intestinal tissue and the Vδ2 population resides in the peripheral blood, carrying out their distinctive functions. From our analysis, it can be seen that Vδ2 T cells, the major subtype of γδ T cells in the blood circulatory system, contributed more than half of the γδ T cells in the younger age group (< 45 years old) (Supplementary Fig. 2). The number gradually decreased below 50% after 45 years of age. This may suggest that Vδ2 might not be the dominant γδ T cell population post mid-age in a healthy individual.

### NK Cells Increase in the Elderly Population

In addition to T cells, changes in the immune parameters of NK cells are also an important indicator of immune age. NK cells primarily perform killing functions and mediate cytotoxicity during the anti-infectious and anti-tumour immune responses (Poli et al. 2009). The percentage of NK cells tends to be higher at a younger age and sharply decreases between 25 and 35 years. It is followed by a relatively steady increase between 35 and 60 years. Finally, there is a sharp increase between 55 and 70 years. The trend of change in percentage of NK cells was similar in males and females; however, approximately 1% lower NK cells were found in females (7.2–10.5%) compared with that in males (7.9–11%) in most age groups, except in the elderly above 70 (Supplementary Fig. 3).

### Immune-Ageing Random Forest Machine-Learning Modelling

To successfully integrate 36 immune variable datasets, we first needed to recognise the biological uniqueness amongst these variables. Amongst the 36 immune variables, four pairs of variables were found to be highly related (Supplementary Fig. 3a). Amongst these four highly similar pairs, one was selected from each pair. Thus, 32 immune variables were selected for immune-ageing modelling (Supplementary Fig. 3b).

**Fig. 3:**
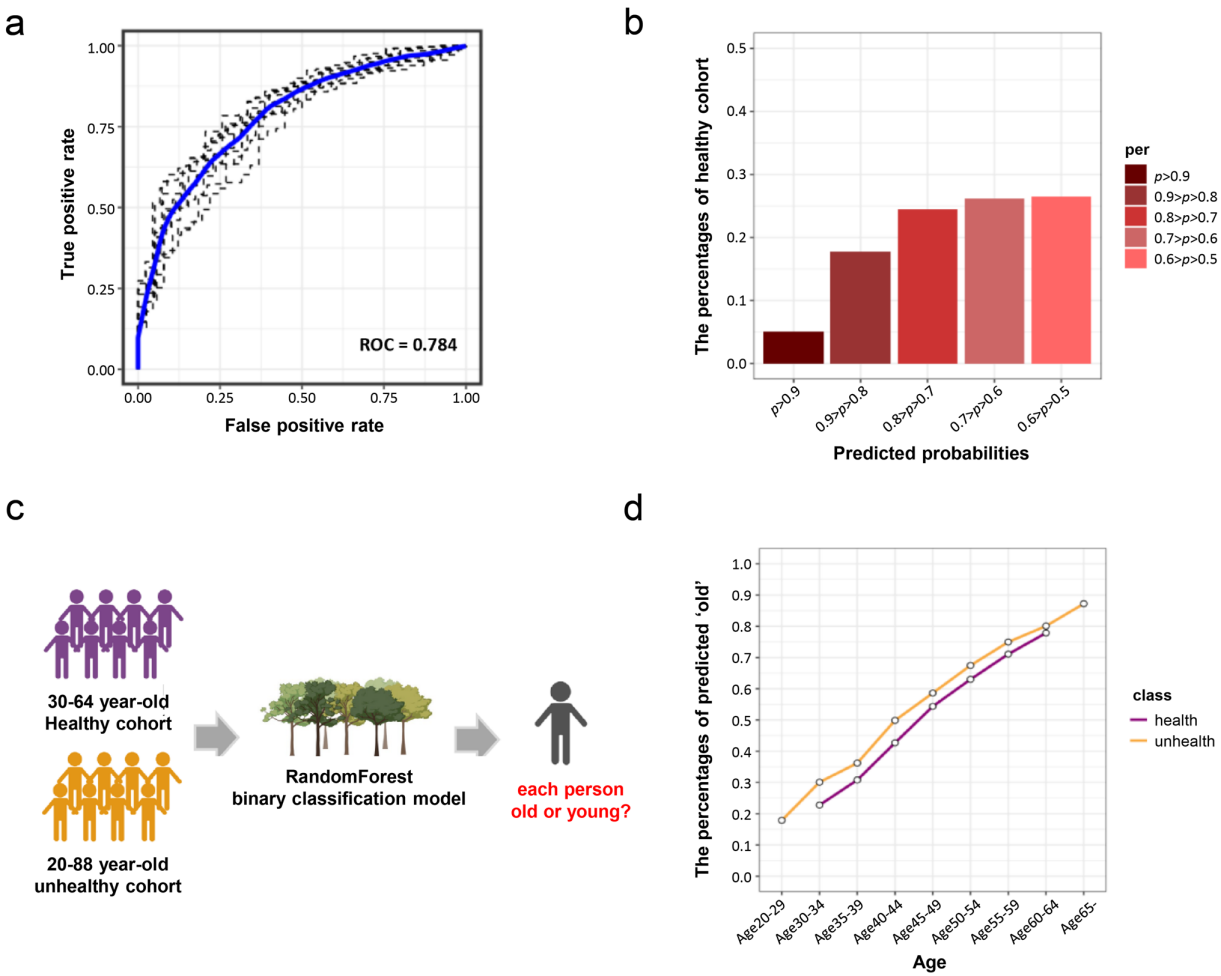
Summary of the immune age random forest machine-learning model. **a** ROC of the random forest machine-learning model using 32 selected immune cell subtype population size from the healthy young (20–29 year-old) and old (≥ 65 year-old) donors. **b** Histogram of the predicted probability as old using the random forest model for the mid-aged healthy (30–64 year-old) donors. **c** Schematic of immune age prediction using the random forest model. **d** Proportion of individuals in each 5-year interval predicted as old using the random forest model for the mid-aged healthy (30–64 year-old) and unhealthy (20–88 year-old, with chronic diseases) donors

To establish immune-ageing RF modelling, two populations aged between 20 and 29 and that of health donors, older than 65 years, who did not have known diseases, were selected to build the model. This training set of data generated a ROC curve with a value of 0.784 (Fig. 3a). The model was further validated with the 30–64 year-old cohort, and the histogram showed the predicated probability as ‘old’ by the model for mid-aged healthy donors (Fig. 3b).

Thereafter, we further applied a cohort of 20–88 year-olds with known diseases to the model (Fig. 3c); we observed that the prediction trends in the healthy and disease cohorts were very similar. The unhealthy cohort has an approximate 10% higher probability of being predicted as ‘old’ compared with the healthy individuals in the same age group. Although the differences appeared to get smaller after 50 years of age (Fig. 3d), this may suggest that the same age group people with known diseases tend to have an ‘older immune age’ compared with their actual ‘biological age’.

**Fig. 4:**
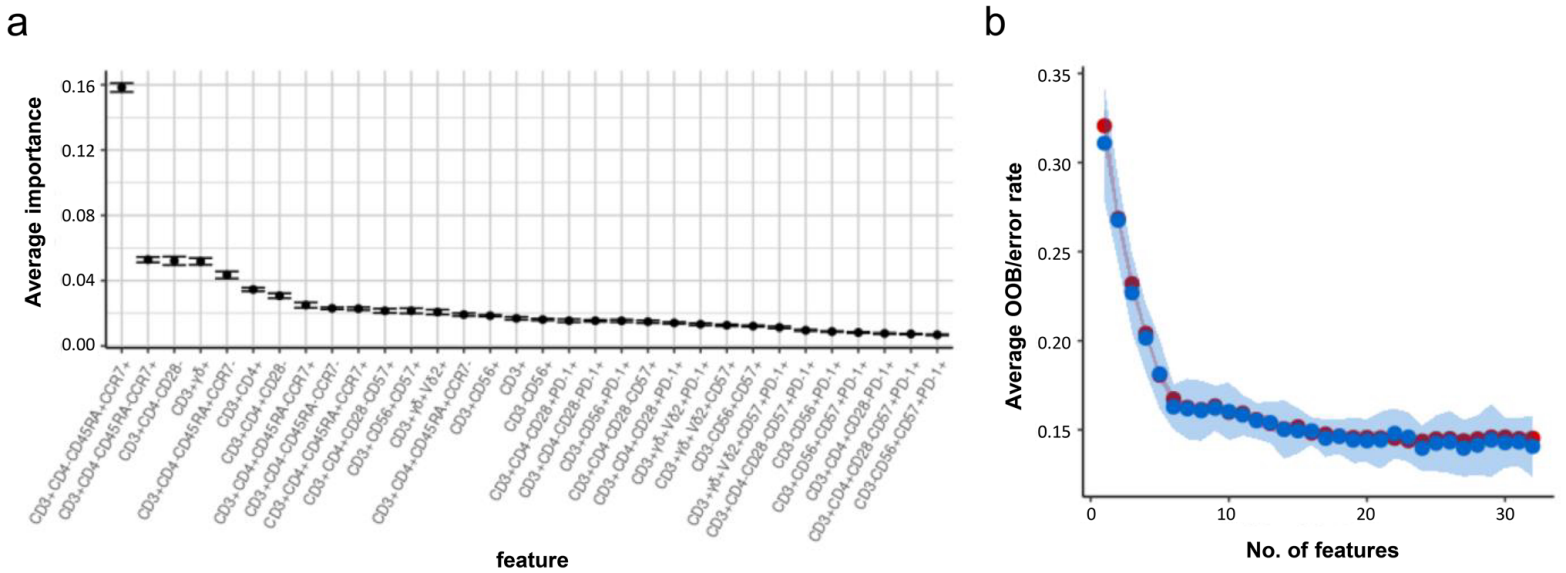
Importance ranking for the immune cell subtype population size. **a** Importance of features calculated using the random forest model for the 32 selected immune cell subtypes. **b** The average out-of-bag (OOB) and error rate change with increasing number of features used in the random forest model. For each iteration of model building, the features were added according to the decreasing order of the feature importance. The model stabilises after around the top six most important features were used in the model

The 32 immune subtypes were further selected for feature importance calculation (Fig. 4a). In agreement with the linear analysis of the previous age group, CD8 T cell subsets were found to have the most significant impact in RF modelling. Naïve CD8 T cells with a score of 0.16 were the highest average importance amongst selected features. CD8-differentiated subtypes, including central memory CD8 and terminally differentiated CD8 T cells were amongst the top features contributing to immune age prediction modelling. Notably, γδ T cells were amongst the top five importance features in the feature selection. As expected, PD-1-associated populations were found to have no impact on the modelling analysis.

We further applied an out-of-bag error rate test to identify the minimum number of features required to establish a reasonable predicted model. As seen in Fig. 4b, with the top six most important features (naïve CD8 T cells, central memory CD8 T cells, CD28^+^CD8 T cells, γδ T cells, terminally differentiated CD8 T cells, CD4 T cells) the model became stabilised. A further 10 features (total of 16 highest ranked important features) allowed the model to become the most accurate with the lowest error rate = 0.15.

Neural network modelling was used to test the accuracy of our RF modelling analysis. The same grouping approach was used in neural network modelling (Supplementary Fig. 4a, b). When compared to the RF analysis, the ROC rate is marginally lower, with an ROC = 0.7, rather than 0.78 (Supplementary Fig. 4c). Another important feature when using this modelling was that the percentage of the healthy cohort has a higher chance of being considered ‘old’ (Supplementary Fig. 4d). In the neural network modelling analysis, stabilisation was reached when the top 17 features were used.

### Analysis of the Absolute Count of T and NK Cells

**Fig. 5:**
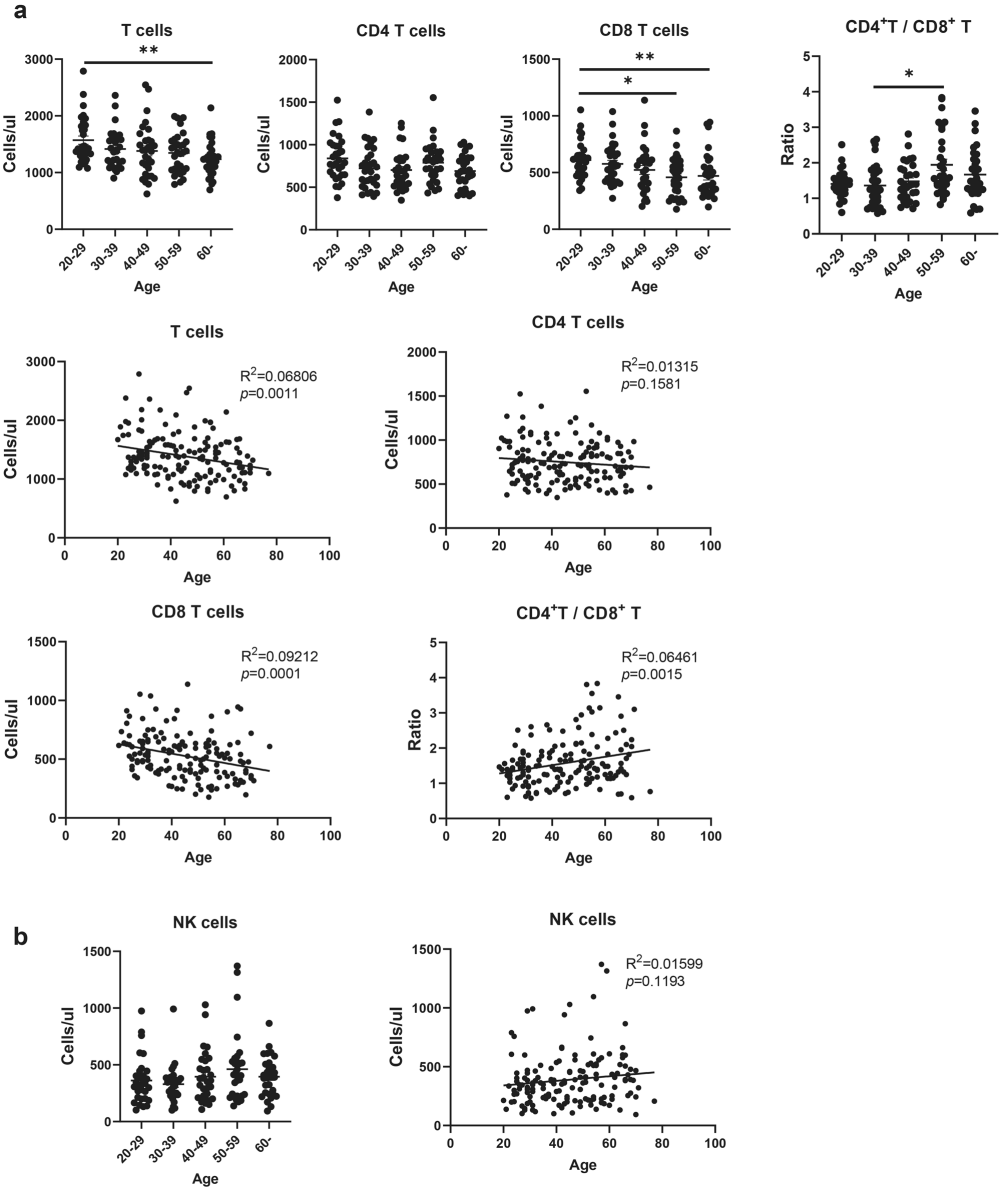
Absolute count for T and NK cells. **a** Absolute count for T, CD4, and CD8 T cells. **b** Absolute count for NK cells

We further enrolled 153 healthy individuals, aged between 20 and 77 years (20–29 years: 31; 30–39 years: 31; 40–49 years: 30; 50–59 years: 31; over 60 years: 30) to detect the absolute count of T and NK immune cell subsets. The absolute T cell count declined with ageing, showing an inverse correlation between age and absolute T cell count. In agreement with the results of our percentage analysis, a decrease was found in the absolute count of CD8 T cells, but not in the CD4 T cell population, with ageing. Furthermore, the CD4/CD8 T cell ratio was positively correlated with age, and the ratio was the highest in the 50–59 years group (Fig. 5a). Although the NK cell proportion was increased in the older people, the absolute count was relatively stable in all the different age groups (Fig. 5b).

## Discussion

Immune ageing, which is defined as the decline in immune function with ageing, greatly impacts the susceptibility to various foreign pathogens, and possibly increases the chances of getting certain types of cancers (Mittelbrunn and Kroemer 2021; Mogilenko et al. 2021b). Several studies have focussed on the correlation of different immune subtypes with the onset of different diseases as well as with clinical outcomes, and on the mechanisms for the decline in the immune function (Sun et al. 2022). However, the immune cell composition in different age groups and their dynamic changes are still poorly understood.

It is of great importance to reflect dynamic changes in the human body through the intuitive research method of cell counting. Lymphocytes primarily include T cells, B cells, and NK cells. As other studies and our preliminary data suggest, B cells do not fluctuate greatly through ageing (Frasca et al. 2022; Mogilenko et al. 2021a). Here, we analysed the proportions of T- and NK-cell subsets in the circulating peripheral blood of a large cohort of Chinese adults in different age groups. Through data analysis, we further applied machine learning to build an immune-ageing model. Using this model, we could hopefully identify the most influencing immune-ageing factors and predict the immune ageing of individuals. In our cohort study, we identified that the proportions of most age-affected immune cells started to decline after 35 years. Around these age inflection points, the proportion of immune cells, including that of CD4^+^ T cells, CD8^+^ T cells, and γδ T cells, changed considerably and was generally considered to be related to the occurrence of cancers and some immunodeficiency diseases (Joosten and Ottenhoff 2008; Minami et al. 2006; Ribot et al. 2020; Strizova et al. 2020). Therefore, changes in immune functions, especially in the proportion of immune cells, might partially elucidate the causes of these diseases.

T cell differentiation is an important process that highly affects the immune responses and is associated with various immunological and haematological disorders (Xia et al. 2022). T cell differentiation occurs when naïve T cells enter programme development and further differentiate into central memory T cells, effector memory T cells, and memory stem like T cells upon antigen stimulation (Luo et al. 2022). The detection of changes in circulating T cells can be valuable in monitoring the onset and progression of disease. Therefore, it is important to establish the reference range for differentiated subsets. In our analysis, which is in agreement with the findings in other studies, the number of naïve T cells decreased with ageing. It is known that the thymus output declines with ageing, which is probably directly responsible for the number of naïve T cells. Notably, in our large cohort modelling analysis, we identified that the effects of ageing on CD4 T cell differentiation are far less than those on CD8 T cell differentiation. One reason might be that the exhaustion of CD8 naïve T cells is greater than that of CD4 cells. The underlying mechanisms is yet to be studied.

The influence of sex differences on the human body has been of great concern. Currently, sex differences have mainly been focussed upon in the field of genomics (Fragiadakis et al. 2022). Owing to the vast range of genetics, transcriptomics, and genomics studies, it is difficult to investigate changes in immune functions from the perspective of genomics in a targeted manner. However, by comparing macroscopic cell-level monitoring, the same marked differences could be obtained with less work. Sex could substantially affect the proportion of different types of immune cells, including that of CD4^+^ T cells, CD8^+^ T cells, NK cells, and T cell differentiated populations.

Previous studies have demonstrated that cells with faster differentiation and more complex functions are more likely to accumulate DNA damage and mutations. T cells are derived from haematopoietic stem cells, which have a specific structure. T cells can accumulate DNA damage and mutations over time. Conversely, increased DNA damage and mutations may further induce the T cell failure (Barnett and Barnett 1998). As innate immune cells, T cell-mediated responses are involved in anti-tumour, anti-infection, and antigen recognition processes. The percentages of these cells are greatly affected by age and sex. Different subsets showed unique trends and age inflection points for immune functions, which might be attributable to the differences in hormone levels of each sex and age group (Krzych et al. 1981). As a bridge between innate and adaptive immune responses, innate T cells have a spontaneous activation phenotype and an Major Histocompatibility Complex (MHC)-independent antigen recognition process, which shows the unique function and potential application value of these T cells.

Human immune age may be different from chronological age. In addition to T cells, changes in NK-cell immune parameters are also an important indicator of immune age. NK cells mainly perform killing functions and mediate cytotoxic abilities in the process of anti-infectious and anti-tumour immune responses (Poli et al. 2009). NK cells and their subsets were found to be relatively stable. In a previous study, it was shown that age shaped the composition and function of T cells. For example, in a cohort of Gambian individuals, type 2 innate lymphoid cells (ILC2) frequencies and absolute counts declined from childhood until about 30 years of age, and type 3 innate lymphoid cells (ILC3) cells frequencies and absolute counts declined steadily across the life course (Darboe et al. 2020). In another study on Gambian individuals, the CD8 T cell population was more differentiated in Cytomegalovirus (CMV)-infected infants than in uninfected at 12 months-old infants (Miles et al. 2007). In addition, NK-cell maturation and differentiation is highly age dependent. A study in the Gambian population showed that increasing frequencies of NK cells with age were associated with increased proportions of CD56^dim^ cells expressing the differentiation marker CD57 and expansion of the NK cell Group 2 isoform C (NKG2C^+^) subset (Goodier et al. 2014).

Although the above results partially illustrate the impact of age on differences in immune parameter by sex within the Chinese population, there are still some constraints in this study. First, the percentage of samples above 70 years old was relatively small, and this might imply that the dataset may cause a population bias. The implications on the percentages and change trends for immune cells in individuals who are not in this age range are rather limited. In addition, 65,532 individuals were initially involved in the study, but only 43,096 were included in the final analysis; thus, the final analysis results might represent only a part of the population. Second, the design of the experiments also had some limitations. Although the above results were representative, the functional assessment was not performed for these cohorts. Third, the fluorescence detected using flow cytometry used in this study was low and cannot fully reflect high scientific significance. Our study focussed on the comprehensive and rapid detection of human immune functions, which has an objective importance in clinical application.

To our knowledge, this study included the largest cohort for the most comprehensive integrated evaluation of T and NK cells. In our study, ageing was responsible for certain immune cell subsets dynamics. For instance, naïve CD8^+^T and naïve CD4^+^T cells exhibited a decline across ageing, which was consistent with the study in a Western European ancestry (Patin et al. 2018) and Belgian population (Carr et al. 2016). In addition, the number or percentage of CD4^+^naïve and CD8^+^naïve cells decreased in the report by Calogero Caruso and co-workers (Caruso et al. 2022). Moreover, the senescent CD8^+^T cells (CD3^+^CD8^+^CD28^−^) and terminal differentiation CD8^+^T cells (CD3^+^CD8^+^CCR7^−^CD45RA^+^) showed an increase with age. In agreement with over nine years of high-dimensional longitudinal monitoring, their cross-sectional analysis showed that the CD8^+^CD28^−^T cell subset increased with age (Alpert et al. 2019). A study of the Sicilian population showed a significant increase in the percentage of CD3^+^CD8^+^CCR7^−^CD45RA^+^T cells with age (Ligotti et al. 2021). Our data show that the proportion of γδ T cells decreased with age after 35 years, whereas results of a previous study indicated that the percentage of γδ T cells remained unchanged with age (Ligotti et al. 2021). Their result indicated that the percentage of NK cells positively correlated with age, whereas our data show that the percentage of NK cells fluctuated greatly with age.

The evaluation of immune age was mainly based on changes in immune parameters of these cell types. The results of our current study indicate that human immune age might be different from chronological age. Using machine learning, we identified immune factors that are affected the most during ageing and built a model for the prediction of immune ageing. Our research not only revealed the impact of age on differences in immune parameters by sex within a Chinese population, it also provides new insights for the monitoring and prevention of some diseases in clinical practice, which has social benefits.

## Conclusion

In summary, this study depicts the dynamic change of T lymphocyte subsets and NK-cell subsets by ageing and sex, and built a model for prediction of immune ageing. Human immune age might be different from chronological age. Further investigation into immune age might be warranted to provide new insights to healthy status.

## Supplementary Information

Below is the link to the electronic supplementary material.

Supplementary file1 (PDF 2138 KB)

## Data Availability

The datasets used for this study are available upon request from the corresponding authors.

## Abbreviations

NK cell: Natural killer cell
CD: Cluster of differentiation
CCR7: CC-chemokine receptor 7
PD-1: Programmed cell death protein 1
RF: Random forest
ROC: Receiver operating characteristic
TNK: T natural killer
