# Supplementary material for: Immune-Ageing Evaluation of Peripheral T and NK Lymphocyte Subsets in Chinese Healthy Adults
Source: Phenomics. 2023 May 23;3(4):360–74. doi: 10.1007/s43657-023-00106-0 (PMC10425318; doi:10.1007/s43657-023-00106-0)
Supplement: Supplementary file 1 — Supplementary file1 (PDF 2138 KB) [file 43657_2023_106_MOESM1_ESM.pdf]

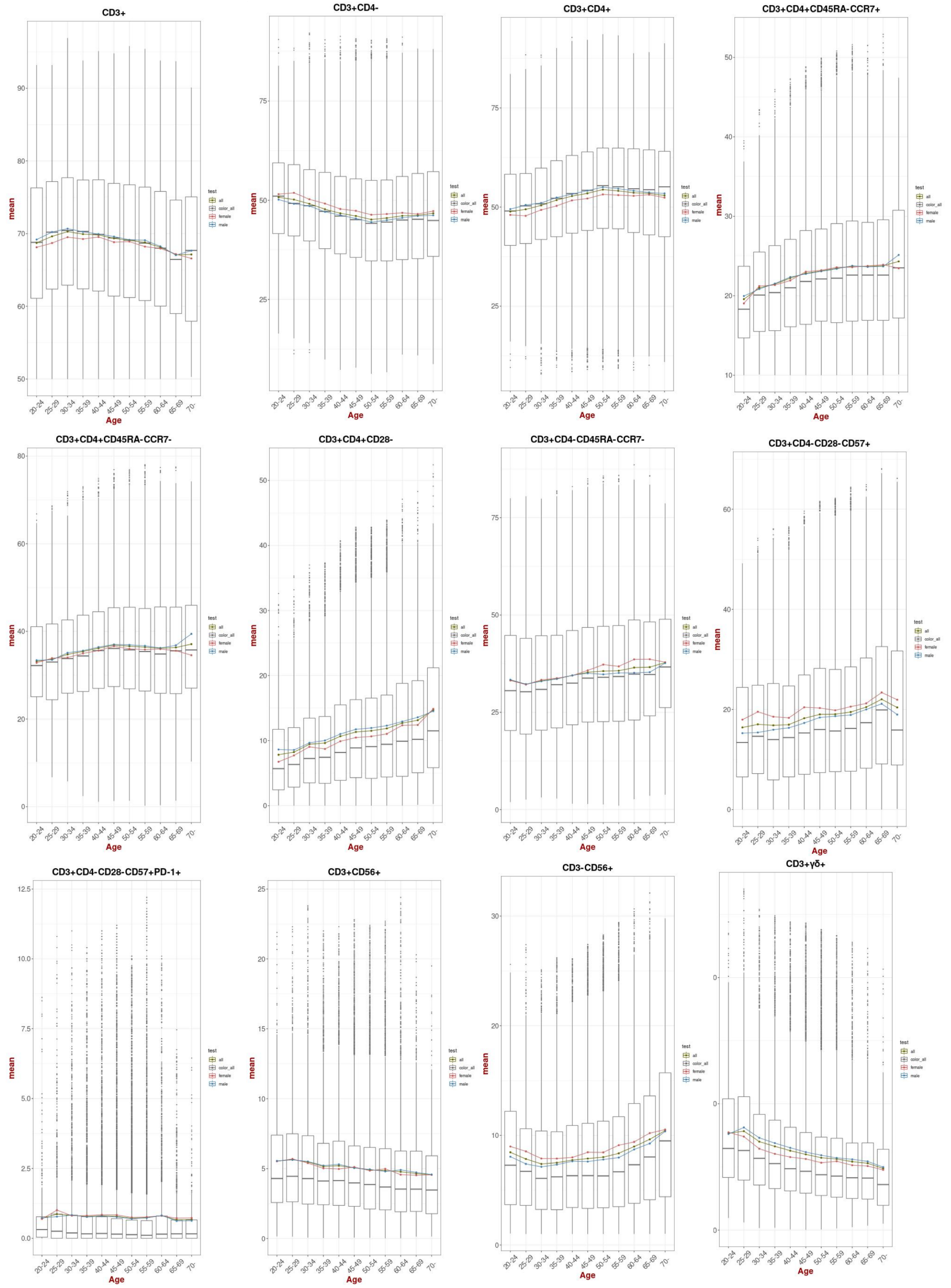

Figure S1 The value-age linear regression of selected immune cell subtype population size by each 5 years age interval. The red, blue and green dots represent the mean values for female, male and all donors, respectively.

Figure S2

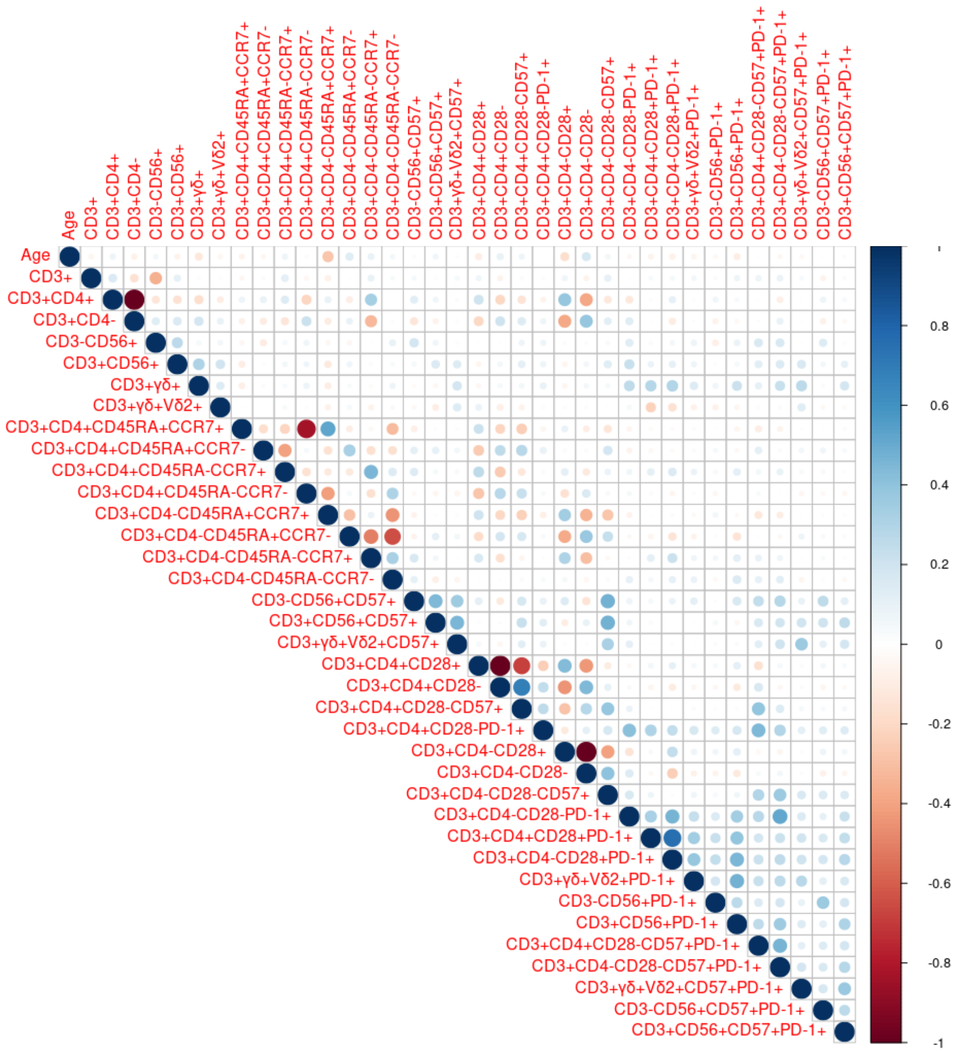

Figure S2 The correlation of different immune subtypes

Figure S3

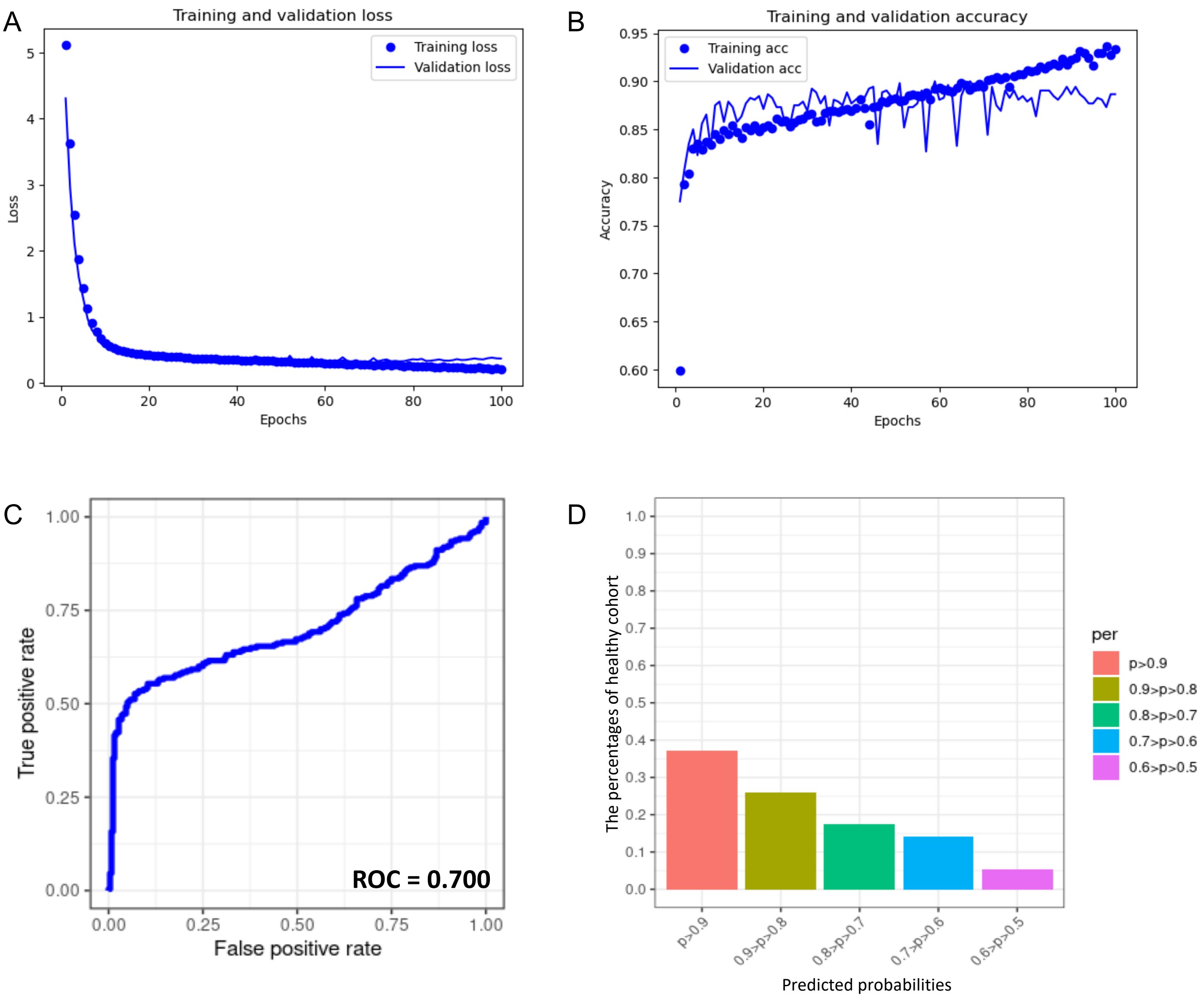

Figure S3 Summary of the immune-age neural network machine-learning models.
(A-B) The loss (A) and accuracy (B) of training and validation dataset change with adding the iteration in building neural network machine-learning model.
(C) ROC of the neural network model using 32 selected immune cell subtype population size from the healthy young (20-29 year-old) and old (≥65 year-old) donors.
(D) Histogram of the predicated probability as old by the neural network model for the mid-age healthy (30-64 year-old) donors.

Table. S1

	RegionGroups (year-old)		
	North	South	
CD3+	72.01±10.16	68.97±9.91	**
CD3+CD4+	53.91±14.28	53.12±14.3	**
CD3+CD4-	45.74±14.24	46.39±14.31	*
CD3-CD56+	7.78±5.84	8.05±5.85	*
CD3+CD56+	5.22±3.87	5.02±3.94	**
CD3+γδ+	5.54±4.09	6.07±4.49	**
CD3+γδ+Vδ2+	45.86±24.62	50.52±25.02	**
CD3+CD4+CD45RA+CCR7+	31.06±13.88	32.06±13.88	**
CD3+CD4+CD45RA+CCR7-	8.97±6.25	8.69±6	*
CD3+CD4+CD45RA-CCR7+	23.85±8.39	22.99±8.29	**
CD3+CD4+CD45RA-CCR7-	36.13±13.06	36.25±12.95	ns
CD3+CD4-CD45RA+CCR7+	19.05±12.57	21.41±13.48	**
CD3+CD4-CD45RA+CCR7-	37.97±16.46	39.09±17.14	**
CD3+CD4-CD45RA-CCR7+	5.05±3.32	4.56±3.28	**
CD3+CD4-CD45RA-CCR7-	37.93±16.82	34.94±16.37	**
CD3-CD56+CD57+	42.05±24.81	35.92±23.49	**
CD3+CD56+CD57+	34.33±21.65	31.2±20.81	**
CD3+γδ+Vδ2+CD57+	16.8±18.92	16.89±19.48	ns
CD3+CD4+CD28+	88.32±9.14	88.86±9	**
CD3+CD4+CD28-	11.66±9.12	11.11±8.98	**
CD3+CD4+CD28-CD57+	6.6±6.17	5.4±5.57	**
CD3+CD4+CD28-PD-1+	0.14±0.28	0.13±0.27	ns
CD3+CD4-CD28+	53.85±16.99	55.43±17.05	**
CD3+CD4-CD28-	46.14±16.99	44.56±17.04	**
CD3+CD4-CD28-CD57+	23.29±15.05	18.46±13.86	**
CD3+CD4-CD28-PD-1+	0.69±0.98	0.62±0.95	**
CD3+CD4+CD28+PD-1+	5.6±6.03	5.14±6	**
CD3+CD4-CD28+PD-1+	4.09±3.76	3.73±3.66	**
CD3+γδ+Vδ2+PD-1+	5.46±6.34	5.15±6.38	*
CD3-CD56+PD-1+	1.17±1.66	1.13±1.67	ns
CD3+CD56+PD-1+	4.33±4.69	4.22±4.94	ns
CD3+CD4+CD28-CD57+PD-1+	0.83±1.92	0.42±1.36	**
CD3+CD4-CD28-CD57+PD-1+	0.99±1.75	0.74±1.53	**
CD3+γδ+Vδ2+CD57+PD-1+	1.18±2.06	1.23±2.05	ns
CD3-CD56+CD57+PD-1+	0.42±0.66	0.39±0.67	*
CD3+CD56+CD57+PD-1+	1.54±2.23	1.47±2.35	ns
